# Health care provider practices in diagnosis and treatment of malaria in rural communities in Kisumu County, Kenya

**DOI:** 10.1186/s12936-022-04156-z

**Published:** 2022-04-22

**Authors:** Wilfred Ouma Otambo, Julius O. Olumeh, Kevin O. Ochwedo, Edwin O. Magomere, Isaiah Debrah, Collins Ouma, Patrick Onyango, Harrysone Atieli, Wolfgang R. Mukabana, Chloe Wang, Ming-Chieh Lee, Andrew K. Githeko, Guofa Zhou, John Githure, James Kazura, Guiyun Yan

**Affiliations:** 1 Department of Zoology, Maseno University, Kisumu, Kenya; 2 International Centre of Excellence for Malaria Research, Tom Mboya University College of Maseno University, Homa Bay, Kenya; 3 Department of Biology, Faculty of Science and Technology, University of Nairobi, Nairobi, Kenya; 4 Department of Biochemistry and Molecular Biology, Egerton University, Njoro, Kenya; 5 West Africa Centre for Cell Biology of Infectious Pathogen, Department of Biochemistry, Cell and Molecular Biology, University of Ghana, Accra, Ghana; 6 Department of Biomedical Sciences and Technology, Maseno University, Kisumu, Kenya; 7 Depatment of Population Health and Disease Prevention, University of California, Irvine, CA USA; 8 Centre for Global Health Research, Kenya Medical Research Institute, Kisumu, Kenya; 9 Centre for Global Health and Diseases, Case Western University Reserve, Cleveland, OH USA

**Keywords:** Malaria, Blood smear, Kenya, Treatment guidelines, Misdiagnosis, Presumptive treatment

## Abstract

**Background:**

Accurate malaria diagnosis and appropriate treatment at local health facilities are critical to reducing morbidity and human reservoir of infectious gametocytes. The current study assessed the accuracy of malaria diagnosis and treatment practices in three health care facilities in rural western Kenya.

**Methods:**

The accuracy of malaria detection and treatment recommended compliance was monitored in two public and one private hospital from November 2019 through March 2020. Blood smears from febrile patients were examined by hospital laboratory technicians and re-examined by an expert microscopists thereafter subjected to real-time polymerase chain reaction (RT-PCR) for quality assurance. In addition, blood smears from patients diagnosed with malaria rapid diagnostic tests (RDT) and presumptively treated with anti-malarial were re-examined by an expert microscopist.

**Results:**

A total of 1131 febrile outpatients were assessed for slide positivity (936), RDT (126) and presumptive diagnosis (69). The overall positivity rate for *Plasmodium falciparum* was 28% (257/936). The odds of slide positivity was higher in public hospitals, 30% (186/624, OR:1.44, 95% CI = 1.05–1.98, *p* < 0.05) than the private hospital 23% (71/312, OR:0.69, 95% CI = 0.51–0.95, *p* < 0.05). Anti-malarial treatment was dispensed more at public hospitals (95.2%, 177/186) than the private hospital (78.9%, 56/71, *p* < 0.0001). Inappropriate anti-malarial treatment, i.e. artemether-lumefantrine given to blood smear negative patients was higher at public hospitals (14.6%, 64/438) than the private hospital (7.1%, 17/241) (*p* = 0.004). RDT was the most sensitive (73.8%, 95% CI = 39.5–57.4) and specific (89.2%, 95% CI = 78.5–95.2) followed by hospital microscopy (sensitivity 47.6%, 95% CI = 38.2–57.1) and specificity (86.7%, 95% CI = 80.8–91.0). Presumptive diagnosis had the lowest sensitivity (25.7%, 95% CI = 13.1–43.6) and specificity (75.0%, 95% CI = 50.6–90.4). RDT had the highest non-treatment of negatives [98.3% (57/58)] while hospital microscopy had the lowest [77.3% (116/150)]. Health facilities misdiagnosis was at 27.9% (77/276). PCR confirmed 5.2% (4/23) of the 77 misdiagnosed cases as false positive and 68.5% (37/54) as false negative.

**Conclusions:**

The disparity in malaria diagnosis at health facilities with many slide positives reported as negatives and high presumptive treatment of slide negative cases, necessitates augmenting microscopic with RDTs and calls for Ministry of Health strengthening supportive infrastructure to be in compliance with treatment guidelines of Test, Treat, and Track to improve malaria case management.

## Background

Malaria remains a major public health concern and a leading cause of morbidity and mortality in the tropics. 241 million malaria cases were reported globally in 2020, with the African region accounting for ~ 95% of all cases [1]. Seventy percent of Kenya’s 47 million people are at risk of malaria with the western Kenya region bearing the highest malaria burden [2]. Challenges to malaria control in Kenya include not only mosquito vector resistance to insecticides and increasing outdoor biting behaviour, but also socioeconomic and logistical variables, such as poverty and uneven access to appropriate prevention, diagnostic and treatment regimens [3]. Children under age five years and pregnant women are among the most vulnerable demographic groups to malaria morbidity [4, 5]. School age children and adults have a high prevalence of asymptomatic *Plasmodium falciparum* infection (subclinical malaria), and thus serve as the main reservoir of gametocytes that sustain transmission [6].

The primary strategy for malaria treatment is timely and accurate diagnosis followed by effective treatment. According to WHO guidelines, all patients suspected of having malaria should have blood stage infection confirmed by microscopic inspection of blood smears or a malaria-specific Rapid Diagnostic Test (RDT) before anti-malarial drug treatment. Presumptive diagnosis based on clinical features and history in the absence of laboratory confirmed blood stage infection is appropriate only when expert microscopic inspection of blood smears or RDTs are not available [7]. Artemisinin-based combination therapy (ACT), such as artemether-lumefantrine, (AL), is recommended for treatment of non-life threatening uncomplicated falciparum malaria while parenteral artesunate is recommended for severe malaria [7]. Nevertheless, treatment with anti-malarial drugs still occurs in some patients in absence of or with negative diagnostic tests [8].

The malaria burden in Kenya remains high despite efforts by the Ministry of Health to scale up various public health interventions, such as long-lasting insecticidal nets and indoor residual spraying of insecticides [9–11]. According to the Kenyan National Malaria Treatment Guidelines, all suspected malaria cases should be tested for parasites using microscopy or RDTs to ensure that patients with fever from other causes are managed appropriately and that treatment is directed toward patients with confirmed malaria infection.[12]. Although algorithms of clinical symptoms indicative of malaria are readily available along with expertise in reading blood smears and malaria RDTs [13], health care providers may still rely on presumptive clinical diagnosis [14]. This is problematic since, for example, malaria symptoms such as fever, prostration, and myalgia are similar to those of other common infectious diseases caused by viral and bacterial pathogens [15]. In addition, while malaria RDTs are easy to use in remote health facilities, their sensitivity decreases with low parasitaemia, and false-positive results may occur after blood stage parasites have been eliminated by prior treatment with anti-malarial drugs obtained in local community stores outside the health care setting [15]. While microscopy can differentiate between the blood stages of various *Plasmodium* species and provide insight into the parasite biomass that can serve as indicators of malaria severity, reliable reading of blood smears requires skilled microscopists [16].

Performance of blood smears also requires access to well-maintained microscopes and electricity, high quality training and supervision [17]. Inappropriate treatment or failure to treat true blood stage infections can lead to increased healthcare costs [8, 18, 19]. The current study was performed to evaluate health care worker practices regarding clinical and parasitological diagnosis and treatment of malaria in three rural hospitals in western Kenya. Malaria diagnostic and treatment decisions were compared on the basis of retrospective RT-PCR diagnosis of *Plasmodium* infection, a sensitive and specific diagnostic tool that served as an independent “gold” standard confirmatory of infection.

## Methods

### Study design and data collection

This study was conducted in two public hospitals and one private hospital in a rural area of Kisumu County, Kenya near the shores of Lake Victoria. Communities served by public hospital 1, public hospital 2 and the private hospital had resident populations of 16,123, 8,250 and 16,115, respectively. Topographic features of the area include a lowland plain near the basin of Lake Victoria at an elevation of 1100–1200 m above sea level with transition to a rocky slope and highland at 1450–1600 m elevation. Flooding with commensurate increases in the number of mosquito larval habitats generally occur during annual periods of heavy rainfall in April to June and October and November.

Malaria surveys were conducted in hospitals from November 2019 through March 2020. Febrile patients seeking treatment at the health facilities were asked to enroll in the study. A febrile malaria case was defined as an individual with fever (axillary temperature ≥ 37.5 °C) at the time of examination or subjective complaints of fever and other non-specific constitutional symptoms within the previous 24 h [20]. Patients were interviewed using a structured questionnaire related to fever and other malaria symptoms. This information was recorded on digital tablets using REDCap Survey software (Vanderbilt University) that was reviewed daily by team supervisors for quality assurance. Study technicians stationed at the hospitals collected clinical and demographic data from patients as they were referred to the hospital laboratory for diagnostic testing. Symptoms, diagnoses, treatment regimens, and hospitalization were documented, and blood smears were re-examined by an expert microscopist. During the enrollment, outpatient febrile cases were categorized into three groups: (i) patients who were tested for malaria parasite infection by microscopic examination of blood smears at the hospital laboratory and retrospectively by RT-PCR for quality assurance, (ii) those who were tested for malaria parasites by RDT; and (iii) presumptive malaria diagnosis with no blood smear or RDT at the hospital laboratory. This latter group had an axillary temperature ≥ 37.5 °C at the time of examination and a subjective history of fever in the past 24 h. They were triaged by a hospital clinician and prescribed either anti-malarial and/or antibiotic treatment. The International Center of Excellence for Malaria Research (ICEMR) technicians stationed at the health facilities prepared blood smears from the suspect cases that were sent to the ICEMR laboratory for expert microscopic inspection. A total of 1131 febrile cases were enrolled in the study: blood smears and dried blood spots (DBS) on filter paper were collected from 936 patients at hospitals and analysed by microscopy and RT-PCR to determine the slide positivity rate and malaria treatment; 126 cases diagnosed by regular malaria RDT were evaluated for the accuracy of hospital malaria RDT with expert microscopy, whereas 55 presumptively diagnosed cases were evaluated for the accuracy of clinical diagnosis presumptive treatment at health facilities with expert microscopy. A subset of 276 samples were chosen at random from the 936 blood smears and re-examined by expert microscopists for discrepancy in slide reading, misdiagnosis rate, and sensitivity and specificity of the hospital microscopy.

### Processing of blood smears

Finger prick blood smears were collected in duplicate and examined by microscopy. One blood smear was read at the hospital laboratory and the other taken to the ICEMR laboratory in Homa Bay for independent expert reading of blood smears. Thick and thin blood films were stained with 10% Giemsa for 15 min and examined using oil immersion under magnification × 1,000 to identify and count the parasite species. A slide was considered positive if at least one asexual blood-stage *P. falciparum* parasite was identified. Parasite density was determined by counting the number of parasites per 200 leukocytes. The count was then converted to the number of parasites to the equivalent of 8000 leukocytes/μL blood.

### DNA extraction and screening for *Plasmodium falciparum* infection

276 of the 936 dried blood spots were randomly selected for DNA extraction. Chelex resin (Chelex-100) saponin method was used with slight modifications [21]. *Plasmodium* species-specific primers and probes targeting 18S ribosomal RNA were used [22]. PCR reaction volume was constituted as follows; 6 µL of PerfeCTa® qPCR ToughMix™, Low ROX™ Master mix (2X), 0.4 µL each of the forward and reverse species-specific primers (10 µM), 0.5 µL of the species-specific probe, 0.1 µL of double-distilled water and 2 µL of parasite DNA. Thermocycler conditions were set as follows, 50 °C for 2 min, (95 °C for 2 min, 95 °C for 3 s and 58 °C for 30 s) for 45 cycles (QuantStudio™ 3 Real-Time PCR System).

### Data analysis

The data were analysed using SPSS. The Chi-square test was used to test for differences in malaria prevalence and frequency of presumptive treatment. Sensitivity, specificity, positive predicted value, negative predicted value, diagnostic accuracy and Cohen’s kappa statistic were used to calculate adjusted agreement between hospital microscopy and ICEMR microscopy and RT-PCR results. Frequency tables were used to describe categorical variables (counts and percentages).

## Results

### Study participant demographics

A total of 1131 patients who visited the outpatient departments at the three hospitals because of subjective fever and associated non-localizing malaria symptoms agreed to take part in the study. Demographic information of the study participants is summarized in Table 1. There was no significant difference in participants’ sex distributions among the three hospitals (χ^2^ = 1.22; df = 2, *p* = 0.4534); however, there was a higher proportion of female than male participants in all hospitals. Significant differences in the age distributions and occupation of study participants existed amongst the hospitals. A higher proportion of patients with secondary school and college level education sought care at the private hospital than the two public health hospitals (χ^2^ = 449.72; df = 8, *p* < 0.0001).

**Table 1:** Demographics of study populations in three hospitals

Parameters			Hospitals		
Details		Enrollment N (%)	Public 1 N (%)	Public 2 N (%)	Private N (%)
Total enrollment		1131	317	488	326
Blood smear slide examination		936	317	307	312
Malaria rapid diagnosis test		126	0	126	0
Presumptive diagnosis		69	0	55	14
Gender	Male	464 (41.0)	127 (40.1)	195 (40.0)	142 (43.6)
	Female	687 (59.0)	190 (59.9)	293 (60.0)	184 (56.4)
Age	< 5 years	138 (12.2)	66 (20.8)	60 (12.3)	12 (3.7)
	5–15 years	344 (30.4)	96 (30.3)	190 (38.9)	58 (17.8)
	≥ 15 years	649 (57.4)	155 (48.9)	238 (48.8)	256 (78.5)
Education	lliterate	68 (6.0)	6 (1.9)	41 (8.5)	21 (6.4)
	Pre-primary	85 (7.5)	68 (21.5)	9 (1.8%)	8 (2.5)
	Primary	454 (40.1)	99 (31.2)	309 (63.3)	46 (14.1)
	Secondary	349 (30.9)	107 (33.8)	120 (24.6)	122 (37.4)
	College & above	175 (14.8)	37 (11.7)	9 (1.8)	129 (39.6)
Occupation	Farmer	135 (11.9)	39 (12.3)	59 (12.1)	37 (11.3)
	Business person	227 (20.1)	42 (13.2)	120 (24.6)	65 (19.9)
	Office worker	48 (4.2)	15 (4.7)	4 (0.8)	29 (8.9)
	Unemployed	71 (6.3)	15 (4.7)	3 (0.6)	53 (16.3)
	Student	529 (46.8)	138 (43.5)	266 (54.5)	125 (38.3)
	Non-school child	103 (9.1)	66 (20.8)	31 (6.4)	6 (1.8)
	Others	18 (1.6)	2 (0.6)	5 (1.0)	11 (3.4)

### Hospital diagnosis and treatment

Blood smears were prepared and read for 936 febrile cases of the 1,131 outpatient study participants (Table 2). The overall positivity rate for *P. falciparum* was 28% (257/936). The odds of having a positive blood smear was higher in the two public hospitals, 30% (186/624, OR: 1.44, 95% CI = 1.05–1.98, *p* < 0.05) than the private hospital, 23% (71/312, OR: 0.69, 95% CI = 0.51–0.95, *p* < 0.05). There was no significant difference in smear positivity rates between males and females (χ^2^ = 4.263, df. = 2, *p* = 0.1197). In contrast, the smear positivity rate differed according to age. School children aged 5–15 years old had the highest rate (χ^2^ = 45.818, df. = 4, *p* < 0.001).

**Table 2:** *Plasmodium falciparum* Positive Blood Smears Detected in Hospital Laboratories

Hospital		Public 1	Public 2	Private
Total tested		317	307	312
Positive N (%)		98 (30.9)	88 (28.7)	71 (22.8)
Gender N (%)	Male	41/127 (32.3)	41/118 (34.7)	41/136 (30.1)
	Female	57/190 (30.0)	47/189 (24.9)	30/176 (17.0)
Age N (%)	< 5 years	28/66 (42.4)	11/41 (26.8)	2/11 (18.2)
	5–15 years	40/96 (41.7)	54/114 (47.4)	22/58 (37.9)
	> 15 years	30/155 (19.4)	23/152 (15.1)	47/243 (19.3)

Treatment of patients with fever who were blood smear positive or blood smear negative was with AL, antibiotics and analgesics (Table 3). Appropriate anti-malarial treatment was dispensed more frequently at the public hospitals (95.2%, 177/186) than the private hospital (78.9%, 56/71) (χ^2^ = 16.1, df = 1, *p* < 0.0001). Inappropriate anti-malarial treatment, i.e. AL given to blood smear negative patients, was higher at the two public hospitals (14.6%, 64/438) than the private hospital (7.1%, 17/241) (χ^2^ = 8.45, df = 1, *p* = 0.004). More analgesics were given to blood smear negative patients at the private hospital.

**Table 3:** Medications Given to Hospital Blood Smear Negative and Blood Smear Positive Patients

Parameters	Public Hospital 1 and 2		Private hospital		p-value
Blood smear	Positive	Negative	Positive	Negative	
Number of Treatments	n = 186	n = 438	n = 71	n = 241	
AL + analgesic	77 (41.4%)	42 (9.6%)	50 (70.4%)	15 (9.9%)	< 0.0001
Antibiotics + analgesic	5 (2.7%)	273 (62.3%)	2 (2.8%)	76 (50.6%)	< 0.0001
AL + antibiotics + analgesic	100 (53.2%)	22 (5.0%)	6 (17.2%)	2 (1.3%)	< 0.0001
Analgesic only	4 (2.4%) *	101 (23.0%)	7 (6.0%) *	59 (38.8%)	< 0.0001

Antibiotics: Amoxicillin, ciprofloxacin, metronidazole, clotrimazole, * referred to buy antimalarial in the local chemist due to stock-out in Public hospital and patients’ affordability in a private hospital

Treatment of blood smear positive patients who were under 5 years and 5–15 years was higher than study participants older than 15 years. These differences were not statistically significant (*p* = 0.142) (Table 4). There were also no significant differences in prescription of AL treatment among blood smear positive versus blood smear negative study participants stratified according to age. 126 study participants were examined with malaria RDT at public hospital 2, of which 48.4% (61/126) were positive. All these patients were treated with AL.

**Table 4:** Age-related Comparisons of Treatments Given to Hospital Blood Smear Positive and Blood Smear Negative Study Participants

Parameters	< 5 years		5—15 years		≥ 15 years		p-value
Blood smear	Positive	Negative	Positive	Negative	Positive	Negative	
Total N treatments	n = 41	n = 77	n = 116	n = 152	n = 100	n = 450	
AL + analgesic	30 (73.2%)	4 (5.2%)	88 (75.9%)	15 (9.9%)	70 (70.0%)	26 (5.8%)	< 0.0001
Antibiotics + analgesic	1 (2.4%)	47 (61.0%)	1 (0.9%)	76 (50.6%)	1 (1.0%)	188 (41.8%)	0.059
AL + antibiotics + analgesic	9 (22.0%)	2 (2.6%)	20 (17.2%)	2 (1.3%)	18 (18.0%)	16 (3.6%)	0.356
Analgesic only	1 (2.4%)*	24 (31.2%)	7 (6.0%)*	59 (38.8%)	11 (11.0%)*	220 (48.9%)	< 0.0001

Antibiotics: Amoxicillin, ciprofloxacin, metronidazole, clotrimazole, * referred to buy antimalarial in the local chemist due to stock-out in the hospital

### Presumptive diagnosis of malaria

Sixty-nine of the 1131 febrile cases were diagnosed as having malaria without the performance of an appropriate laboratory diagnostic test. This occurred during a period of labour disputes and strikes that resulted in the absence of laboratory technicians. Blood smears from 55 of these cases were prepared and examined by an independent expert ICEMR microscopist to determine whether they had malaria infection. Fourteen of 55 patients had a positive blood smear; nine were appropriately given AL. Forty-one of 55 were blood smear negative; 26 were inappropriately treated with AL.

### Sensitivity and specificity of malaria diagnosis and treatment based on hospital microscopy results and real time PCR as the standard reference

The sensitivity and specificity of malaria diagnosis were determined using 276 blood samples diagnosed by microscopic inspection blood smears at the hospital laboratory, 126 samples diagnosed by malaria RDT at public hospital 2, and 55 presumptive (clinical) diagnosis of malaria in comparison to expert microscopy. Diagnosis by malaria RDT was the most sensitive and specific: 73.8% (95% CI = 39.5–57.4) and 89.2% (95% CI = 78.5–95.2), respectively. This was followed by hospital microscopy with a sensitivity of 47.6% (95% CI = 38.2–57.1) and specificity of 86.7% (95% CI = 80.8–91.0). Presumptive diagnosis had the lowest sensitivity and specificity: 25.7% (95% CI = 13.1–43.6) and 75.0% (95% CI = 50.6–90.4), respectively (Table 5).

**Table 5:** Sensitivity and specificity of hospital malaria diagnosis based on expert microscopy

Expert Microscopy results as standard reference	Hospital laboratory Microscopic inspection of blood smear		Rapid diagnostic test		Presumptive diagnosis	
	Positive	Negative	Positive	Negative	Positive	Negative
Positive	49	54	45	16	9	26
Negative	23	150	7	58	5	15
Sensitivity % (95%CI)	47.6 (38.2, 57.1)		73.8 (60.7, 83.8)		25.7 (13.1, 43.6)	
Specificity % (95% CI)	86.7 (80.8, 91.0)		89.2 (78.5, 95.2)		75.0 (50.6, 90.4)	
Positive Predictive Value % (95% CI)	68.1 (56.6, 77.7)		86.5 (76.3, 94.0)		64.3 (35.6, 86.0)	
Negative Predictive Value % (95% CI)	73.5 (67.1, 79.1)		78.4 (67.0, 86.8)		36.6 (22.6, 53.1)	
Diagnostic Accuracy % (95% CI)	72.1 (66.4, 77.3)		81.7 (71.5, 90.9)		43.6 (28.7, 58.5)	
Agreement (Cohen's kappa)	0.37 (0.25, 0.48)		0.63 (0.50, 0.77)		0.006 (0.00, 0.20)	

The inter-reliability reading between the regular RDT and expert microscopy showed a moderate level of agreement (kappa = 0.633; 95% CI = 0.50–0.77, *p* < 0.0001). Inter-reliability between hospital laboratory microscopy and independent expert microscopy was fair (kappa = 0.37; 95% CI = 0.25–0.48, *p* < 0.0001). The value between hospital presumptive diagnosis and independent expert microscopy was poor (kappa = 0.006; 95% CI = 0.00–0.20, *p* < 0.0001) (Table 5).

Anti-malarial treatment of the cases diagnosed by microscopic inspection of blood smears at hospital laboratories was 95.8% (69/72), 100% for RDT (52/52) and 100% for presumptive diagnosis (14/14) (Table 6). Treatment of true positive cases by RDT and presumptive diagnosis were all at 100%. Using independent expert microscopy as a “gold standard” reference, treatment of true positives was 100% based on RDT and 95.9% (47/49) based on hospital laboratory microscopy. However, clinical diagnosis was 100% (26/26) and hospital microscopy was 95.7% (22/23) effective in treating false positives, but RDT diagnosis was 0% (0/7). Furthermore, RDT diagnosis had the highest non-treatment of the negatives at 98.3% (57/58). Hospital microscopy diagnosis had the lowest at 77.3% (116/150) (Table 6).

**Table 6:** Hospital antimalarial treatment based on expert microscopy

Expert Microscopy results as standard reference	Hospital laboratory Microscopic inspection of blood smear		Rapid diagnostic test		Presumptive diagnosis	
	Positive	Negative	Positive	Negative	Positive	Negative
Positive expert microscopy	49	54	45	16	9	26
Negative expert microscopy	23	150	7	58	5	15
Treatment of hospital diagnosis positives n (%)	69 (95.8)		52 (100)		9 (64.2)	
Treatment of expert microscopy positives n (%)	59 (57.3)		45 (73.8)		22 (62.9)	
Treatment of expert microscopy negatives n (%)	56 (32.4)		7 (10.8)		13 (65.0)	
Treatment of true positive n (%)	47 (95.9)		45 (100)		7 (77.8)	
Treatment of true negative n (%)	34 (16.7)		0		11 (73.3)	
Treatment of false positive n (%)	22 (95.7)		7 (100)		2 (40.0)	
Treatment of false negative n (%)	12 (22.2)		0		16 (61.5)	
Non-treatment of negatives n (%)	117 (67.6)		58 (89.2)		4 (20.0)	

Treatment = Treatment with AL

Additional testing was performed on 276 samples to determine the sensitivity and specificity of hospital laboratory microscopy and independent expert microscopy as well as whether hospital treatment was appropriate using RT-PCR as the gold standard. The hospital microscopy sensitivity of 38.3%, (95% CI = 31.1–45.9) was lower than independent expert microscopy sensitivity of 55.9% (95% CI = 47.9–63.6), but specificity was higher at 91.2% (95% CI = 84.6–95.2) compared to 88.7% (95% CI = 81.1–93.6) (Table 7).

**Table 7:** Sensitivity and specificity of hospital malaria diagnosis based real time PCR

Real time PCR results as standard reference	Hospital laboratory Microscopic inspection of blood smear		Expert Microscopy results	
	Positive	Negative	Positive	Negative
Positive PCR	62	100	90	71
Negative PCR	10	104	13	102
Sensitivity % (95%CI)	38.3 (31.1, 45.9)		55.9 (47.9, 63.6)	
Specificity % (95% CI)	91.2 (84.6, 95.2)		88.7 (81.1, 93.6)	
Positive predictive value % (95% CI)	86.1 (76.3, 92.3)		87.4 (79.0, 92.8)	
Negative predictive Value % (95% CI)	51.0 (44.2, 57.8)		59.0 (51.2, 66.3)	
Diagnostic accuracy % (95% CI)	60.1 (54.1, 66.0)		69.6 (58.1, 79.2)	
Agreement (Cohen's kappa)	0.26 (0.18, 0.35)		0.42 (0.32, 0.51)	

The level of agreement between hospital laboratory microscopy and RT-PCR was lower (kappa = 0.26, 95% CI = 0.18–0.35, *p* < 0.0001) than expert microscopy (kappa = 0.42, 95% CI = 0.32–0.51, *p* < 0.0001) (Table 7).

Treatment of positives using RT-PCR as the standard was lower at 45.7% (74/162) because the majority of positives (55.6%, 90/162) were below the number or parasites detected by microscopic inspection of blood smears (Table 8).

**Table 8:** Hospital antimalarial treatment based on real time PCR

Real time PCR results as standard reference	Hospital laboratory Microscopic inspection of blood smear		Expert Microscopy results	
	Positive	Negative	Positive	Negative
Positive PCR	62	100	90	71
Negative PCR	10	104	13	102
Treatment of positives n (%)	60 (83.3)		55 (53.4)	
Treatment of RT-PCR positives	74 (45.7)		74 (45.9)	
Treatment of RT-PCR negative	21 (18.4)		21 (18.3)	
Treatment of true positive n (%)	54 (87.1)		51 (56.7)	
Treatment of true negative n (%)	15 (14.4)		17 (16.7)	
Treatment of false positive n (%)	6 (60.0)		4 (30.8)	
Treatment of false negative n (%)	20 (20.0)		23 (32.4)	
Non-treatment of real time PCR negatives	93 (81.6)		94 (81.7)	

Treatment = Treatment with AL

**Fig. 1:**
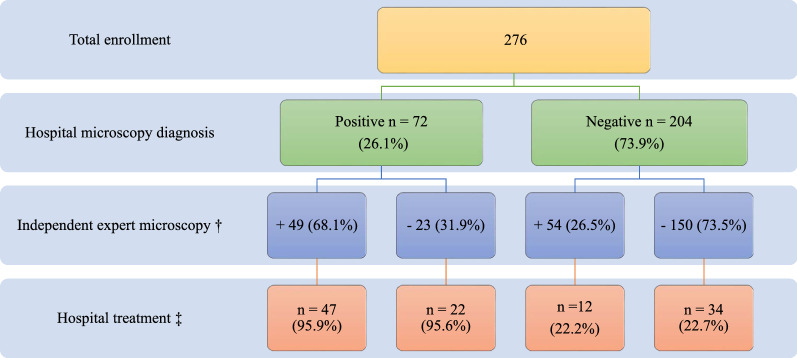
Discrepancy in slide reading and misdiagnosis at health facilities. † + positive,—negative, ‡ All treatments were AL

Misdiagnosis by hospital laboratories occurred in 27.9% of the cases (77/276). Real time-PCR confirmed 4/23 of the 77 misdiagnosed cases as false positive and 37/54 as false negative. Four and one-half percent of the 94 slide positive patients did not receive treatment due to drug stock-outs and were referred to local commercial sources of anti-malarial drugs. This was particularly evident in the patients seeking healthcare at the private hospital. On the other hand, RT-PCR confirmed that 19 of the 34 people with negative blood smears were infected with malaria parasites. 7.3% (15/204) of study participants with negative blood smears and negative PCR results were inappropriately treated with anti-malarial drugs (Fig. 1).

## Discussion

The current study examined healthcare provider malaria diagnosis and treatment practices in 2019–2020 in rural community hospitals in western Kenya. The overall hospital-based malaria infection prevalence by microscopy was 28%, with public hospitals having a higher slide positivity rate (30%) than the private hospital (22.8%). The most sensitive and specific diagnostic method was malaria RDT, followed by hospital microscopy. Presumptive clinical diagnosis without laboratory confirmation was the least sensitive and specific. Appropriate anti-malarial treatment was dispensed more frequently at the public hospitals (95.2%) than the private hospital (78.9%) (*p* < 0.0001). Inappropriate anti-malarial treatment, i.e. AL given to blood smear negative patients, was higher at the two public hospitals (14.6%) than the private hospital (7.1%). Furthermore, RDT diagnosis had the highest rate of non-treatment of negatives (98.3%), while hospital microscopy diagnosis had the lowest rate (77.3%).

According to observations in the current study, hospital microscopy is very specific, but its sensitivity is low. Hospital microscopy has also been found to be insensitive in Ethiopia [30], Tanzania [24], and Cameroon [15]. The low sensitivity of hospital microscopy may have implications regarding the accuracy of diagnosis and treatment. Malaria misdiagnosis and inappropriate treatment continue to be major issues in local health facilities, resulting in anti-malarial and antibiotic overuse [8, 31, 32]. According to the current study, the rate of hospital microscopy misdiagnosis is 28%, which results in inappropriate treatment of patients with AL. Inappropriate treatment with anti-malarials among patients with negative blood smears has been reported elsewhere [33–38]. The possible causes of misdiagnosis could be linked to artifacts misdiagnosed as parasites, lack of equipment maintenance and supervisory or quality-assurance mechanisms in addition to health system infrastructure limitations in which laboratory technicians are under pressure to produce lab diagnoses for several diseases in a short period of time [19]. The current study observed that inconsistency in laboratory technicians' availability at health facilities, insufficient laboratory reagents, as well as power outages and high patient inflow frequently resulted in reduced time of microscopic inspection of blood smears. Taken together, these problems likely contributed to the high misdiagnosis rates.

The odds of testing positive for malaria was 1.4 times higher in the two public hospitals than in the single private hospital where the study was conducted. Appropriate anti-malarial treatment was dispensed more frequently at the public hospitals (95.2%) than the private hospital (78.9%). Malaria infection is common among residents from low-income households, and they commonly seek treatment in public hospitals rather than private hospitals due to lower costs. Low cost treatment in public hospitals may have contributed to the inappropriate anti-malarial treatment, i.e. AL was more frequently given to blood smear negative patients at the two public hospitals (14.6%) than the private hospital (7.1%). However, diagnostic laboratory test fees at health facilities may be prohibitively expensive for resource-poor patients, who in turn may resort to self-treatment by purchasing drugs from loosely regulated local commercial stores. Patients who have received incorrect malaria treatment frequently have other illnesses, particularly bacterial diseases that are not being treated. These comorbidities may increase the overall cost of health care in local communities due to longer hospital stays and repeated outpatient visits [49].

Parasitological diagnosis is recommended for all patients in Kenya suspected of having malaria. Government policy dictates that treatment should not be delayed or denied due to an inability to test for malaria [51, 52]. Under normal circumstances, fever cases are treated with a diagnostic test to confirm malaria infection, followed by the recommended treatment. However, accuracy of misdiagnosis still remains a concern. In the current study, inappropriate presumptively treatment was at 63.4% i.e. patients presumptively treated with the anti-malarial were confirmed negative by an expert microscopist. This is consistent with findings from Vihiga and Kakamega counties in western Kenya where 36% of patients who were presumed positive for malaria by the hospital laboratory did not have the blood stage infection [36]. Such trends have been reported in other health facilities [8, 16, 32, 50]. Labour disputes involving health workers are not uncommon in Kisumu County, resulting in the health facilities' commitment to clinical excellence being compromised.

The effectiveness of malaria intervention strategies is determined by whether people with malaria can easily access and receive appropriate diagnosis and treatment at a reasonable cost. The T3: Test, Treat, Track is a WHO initiative that encourages malaria-endemic countries to test every suspected case of malaria, treat every confirmed case, and track the disease through a timely and accurate surveillance system [55]. The current study findings revealed a high prevalence as well as misdiagnosis and inappropriate treatment of malaria in rural communities in Kisumu County. With the high sensitivity of malaria RDT, hospitals with poor microscopy should augment their diagnostic capability with RDTs to reduce the high proportion of misdiagnosed cases. The study, therefore, recommends strengthening of supportive supervision, monitoring, and evaluation of technicians performing the diagnosis in health facilities in order to fully implement effective malaria case management.

## Limitations

The current study had some limitations. The study was of relatively short duration in 2019 to 2020 and was limited to three major hospitals in rural Kisumu County. The findings may not be generalizable to all hospitals in the County. The majority of patients with fever seek health care at lower level health facilities outside hospitals. Thus, it is possible that the results presented here are not representative of malaria diagnosis and treatment practices in such facilities.

## Conclusion

Misdiagnosis and inappropriate treatment of malaria were found to be a barrier to compliance with national guidelines for malaria management in public and private hospitals in Kisumu County. RDT appeared to be more sensitive and specific than microscopy with specificity within acceptable limits, but insufficient sensitivity. The study recommends that the Ministry of Health invest more in technical training to improve malaria diagnosis capabilities and, for those hospitals that lack microscopists with expertise in reading blood smears, laboratory testing be augmented with RDTs.

## Data Availability

The dataset used in this study is available from the corresponding author upon request.

## Abbreviations

CI: Confidence interval
DBS: Dried blood spots
DNA: Deoxyribonucleic acid
ICEMR: International Center of Excellence for Malaria Research
RDT: Rapid diagnosis test
RT-PCR: Real-time polymerase chain reaction
